# The riverdance of proteins within the cell: a special issue on protein phase separation in biology and diseases

**DOI:** 10.3724/abbs.2023140

**Published:** 2023-07-25

**Authors:** Cong Liu

**Affiliations:** 1 Interdisciplinary Research Center on Biology and Chemistry Shanghai Institute of Organic Chemistry Chinese Academy of Sciences Shanghai 201210 China; 2 State Key Laboratory of Chemical Biology Shanghai Institute of Organic Chemistry Chinese Academy of Sciences Shanghai 200032 China

Over the last decade, the exploration of protein phase separation has gained significant momentum, unfolding as a novel frontier in biological studies. It has revealed a wide array of extraordinary discoveries about how proteins and other biomolecules are dynamically organized in cells, how they function, and the mechanisms behind diseases. This unique issue, titled “Protein Phase Separation in Biology and Diseases”, combines ten reviews that cover a vast range of topics within this burgeoning field. Each review focuses on diverse aspects of phase separation, each making significant progress towards deepening our knowledge and utilization of this critical biological process.

Firstly, it is indispensable to appreciate the intricate methodologies and technological advancements that serve as the bedrock for progress in this field. Ji *et al*. [1] and Li *et al*. [2] meticulously introduce the role of single-molecule and biophysical technologies in delineating the underlying mechanisms and dynamics of phase separation. The magnifying power of these technologies, including single-molecule Förster resonance energy transfer (smFRET), *in vivo* single-molecule imaging technique with single particle tracking (SPT), and atomic force microscopy (AFM), lends a valuable perspective to the minutiae of molecular biophysical properties in biomolecular condensates. Furthermore, they spotlight the interactions between RNA and RNA-binding proteins (RBPs), which are fundamental to the formation of these condensates, highlighting the versatility of these techniques that enable us to overcome limitations of traditional experimental methods.

While these state-of-the-art technologies provide a vantage point into the world of protein phase separation, their significance is fully realized through their applications in the biological sphere. Zheng *et al*. [3] delve into the critical role of liquid-liquid phase separation (LLPS) in cell division, a process integral to life. They detail the way phase separation steers the assembly of membraneless structures during cell division, demonstrating how this process has far-reaching implications in shaping the life cycle of cells. This perspective is broadened by Li *et al*. [4] who articulate the vital influence of LLPS on the transcription cycle. With a focus on transcriptional condensates, they elucidate the dynamic regulation of gene expression in response to intra- and extracellular cues. These reviews affirm the importance of LLPS in maintaining cellular homeostasis and essential physiological function.

Another fascinating area explored is cell signaling and mechanosensing, as discussed by Guo *et al*. [5]. Here, the cellular microenvironment, including membrane curvature, surface topology, tension, and lipid-phase separation, is spotlighted for its role in guiding the assembly of plasma membrane-associated membrane-less organelles (MLOs). This process is critical for signal transduction, the dynamic communication system that regulates cell behavior.

The issue then navigates to the regulatory mechanisms of protein phase separation, with insightful contributions from Li *et al*. [6] and Hou *et al*. [7]. The former dissects the role of small molecules in dictating the properties of condensates and how these molecules can modulate the formation, dissociation, and size of these entities. These findings could serve as the basis for potential novel treatments for condensate-related diseases, linking basic scientific discovery to clinical applications. Complementing this, the latter provides an in-depth exploration of ubiquitination as a key player in phase separation. They shed light on how this post-translational modification, involving the attachment of ubiquitin (Ub) molecules to a protein, modulates the formation of MLOs.

Moving from the mechanisms and regulations of protein phase separation, the issue takes a turn towards the intersection of phase separation and disease. Liu *et al*. [8], Guo *et al*. [9], and Zhang *et al*. [10] shed light on how aberrations in phase separation contribute to pathogenesis. Liu *et al*. [8] particularly discuss the assembly of stress granules, biomolecular condensates that play a protective role during cellular stress, in response to viral infections. Their review provides an understanding of how viruses manipulate host cellular machinery to their advantage. Guo *et al*. [9], on the other hand, explore the association of genetic variations with aberrant protein phase behavior, linking genetic underpinnings to the phase separation machinery in diseases. The concluding review by Zhang *et al*. [10] delves into the role of dysregulated phase separation in cancer and neurodegenerative disorders, with a highlight on potential therapeutic strategies that target pathological condensates.

In conclusion, this special issue enhances our understanding of protein phase separation. It illuminates the role of cutting-edge technologies, the deep connections between phase separation and cellular processes, and the implications of dysregulated phase separation in disease states. Additionally, it indicates the potential for therapeutic interventions by pharmacologically modulating pathological condensates. The reviews in this issue hold the potential to set new directions and offer novel hypotheses in the field of phase separation, establishing a stepping stone for future scientific breakthroughs. Indeed, the world of protein phase separation is as dynamic and complex as the phase-separated condensates themselves. This issue, with its rich ensemble of reviews, fosters a comprehensive understanding of this world, paving the way for potential therapeutic interventions in the future.
